# Paedomorphosis as an Evolutionary Driving Force: Insights from Deep-Sea Brittle Stars

**DOI:** 10.1371/journal.pone.0164562

**Published:** 2016-11-02

**Authors:** Sabine Stöhr, Alexander Martynov

**Affiliations:** 1 Swedish Museum of Natural History, Department of Zoology, Stockholm, Sweden; 2 Zoological Museum, Moscow State University, Moscow, Russia; Laboratoire de Biologie du Développement de Villefranche-sur-Mer, FRANCE

## Abstract

Heterochronic development has been proposed to have played an important role in the evolution of echinoderms. In the class Ophiuroidea, paedomorphosis (retention of juvenile characters into adulthood) has been documented in the families Ophiuridae and Ophiolepididae but not been investigated on a broader taxonomic scale. Historical errors, confusing juvenile stages with paedomorphic species, show the difficulties in correctly identifying the effects of heterochrony on development and evolution. This study presents a detailed analysis of 40 species with morphologies showing various degrees of juvenile appearance in late ontogeny. They are compared to a range of early ontogenetic stages from paedomorphic and non-paedomorphic species. Both quantitative and qualitative measurements are taken and analysed. The results suggest that strongly paedomorphic species are usually larger than other species at comparable developmental stage. The findings support recent notions of polyphyletic origin of the families Ophiuridae and Ophiolepididae. The importance of paedomorphosis and its correct recognition for the practice of taxonomy and phylogeny are emphasized.

## Introduction

A link between individual development (ontogeny) and evolution has been discussed many times since Haeckel's [1] seminal work [2–7]. However only a few decades ago, a special discipline termed as evolutionary developmental biology (evo-devo) was formed [8–10]. Heterochrony has been widely considered as a basic process which is responsible for the evolutionary modifications of ontogeny [11,12] and as a key term to place embryology in a comparative phylogenetic framework [13,14]. Several studies aimed to consider heterochrony in a phylogenetic context [15–18] but in real phylogenetic practice, ontogenetic principles including heterochrony are usually significantly underestimated [19,20]. In taxonomy per se, any consideration of heterochrony is basically absent or insignificant [21], as an intensive search through published literature showed (Table 1). This clearly contradicts the widespread claims that ontogeny is an integral part of evolutionary studies and hence phylogenetics and taxonomy. Here we therefore present an extensive example of heterochronic changes via paedomorphosis among brittle stars and directly link this phenomenon to taxonomic practice.

**Table 1:** Number of publications searched using the Web of Science (Thompson Reuters).

Phylum	Heterochrony	Paedomorphosis	Systematics	Taxonomy	Taxonomy+Ontogeny	Taxonomy+Development
**Arthropoda**	160	87	364651	125408	455	8323
**Chordata**	598	459	153853	54784	687	4584
**Echinodermata**	88	45	9734	2508	87	314
**Mollusca**	139	91	63686	19994	387	1663

Combinations of keywords for several metazoan phyla, including the largest ones (“arthropod*”, “chordat*”, “mollusc*”), taxonomic terms relevant for this study “echinoderm*”, (“systematic*”, “taxonom*”), and ontogenetic terms (“heterochron*”, “paedomorph*”, “ontogen*”, “development*”) for the period 1864–2015 (October 2015).

Heterochronic development, with abbreviated as well as accelerated modes, appears to have played an important role in the evolution of echinoderms. Paedomorphosis, the retention of juvenile characters into adulthood, has been recognized for several groups in early echinoderm evolution, e.g. the Cambrian Eocrinoidea [22], and the post-Palaeozoic Crinoidea [23]. In crinoids, paedomorphosis is considered an important evolutionary mechanism in extinct and extant species [24], and several groups are believed to have a paedomorphic origin [23,25,26]. Paedomorphosis is also known from fossil echinoids [27] and has been argued to be the main force in the evolution of sand dollars [28]. In ophiuroids, paedomorphic development is suspected for some diminutive Carboniferous species (Hotchkiss, personal communication); and the Triassic genus *Nodolanx* has a strongly paedomorphic appearance [29]. Probably the first author who noticed the retention of juvenile features in the adult stage of a brittle star was René Koehler [30], while describing the strongly paedomorphic *Ophiotypa simplex* (Koehler, 1897). However, Koehler simultaneously remarked that such characters are “very primitive”–a typical mistake for many subsequent considerations of secondarily evolved paedomorphic characters as plesiomorphic ones in various taxa [19,31][19]. Matsumoto [32] suggested that some taxa in the family Ophiuridae with a morphology similar to juvenile stages may be paedomorphic. He proposed the (currently unaccepted) subfamily name Ophiomastinae for a group of paedomorphic genera, including *Astrophiura*, *Ophiophycis*, *Ophiomisidium*, *Ophiotypa*, *Ophiomastus*, *Anthophiura*, *Aspidophiura* and *Ophiopyrgus*, all of which are currently placed in Ophiuridae. He also regarded *Ophiomusium* (currently in Ophiolepididae) as a strongly paedomorphic genus. Paedomorphosis has been found to be particularly common in the extant families Ophiuridae and Ophiolepididae [33–35] but this was never investigated or checked on a broadly sampled taxonomic material, nor were the affected structures examined in detail. Peramorphosis, in which the descendant species continues to develop beyond the adult state of its ancestor, has been proposed for Ordovician crinoids [36] and Mesozoic asteroids [37], but not yet for ophiuroids.

Paedomorphic states can be the result of several different types of rate changes in development. Neoteny is the process of slowing down developmental rate with continued growth, whereas progenesis abbreviates the growth period [38]. Neoteny often leads to gigantism, whereas progenesis leads to dwarfism.

These terms have been criticised for poorly defining the evidentiary criteria necessary to distinguish between the modes [39]. For the purpose of this study, these terms may suffice though, because we aim to construct a trajectory of ontogenetic shape changes for the taxa under examination. Progenesis has been suggested as the main cause of paedomorphosis in ophiuroids [34], based on the small size of strongly paedomorphic species. Most species are however expected to show a mosaic of states, some paedomorphic, some peramorphic, some evolving through other mechanisms. Some structures may also be plesiomorphic states that are retained during ontogeny, and never transform into the apomorphic state when development is stopped at an early stage.

Distinguishing paedomorphic states from juvenile ones is not easy, and juveniles of known species have been described as new species. Such was the case with *Ophiuraster patersoni* Litvinova, 1998 which turned out to be the postlarva of *Asteronyx loveni* Müller & Troschel, 1842 [40,41]. Most likely also *Ophiuraster belyaevi* Litvinova, 1998 is the postlarva of another species [41,42]; also several of the species currently included in the genus *Ophiomastus* are likely postlarvae of other species. This clearly highlights the crucial importance of employing consistent ontogenetic principles in taxonomic theory and practice, including the formulation of the special field of *ontogenetic systematics* [20,43]. The present contribution which employs both quantitative and qualitative estimations is thus a further next step in the development of such an approach.

Previous studies assumed paedomorphic development in ophiuroids by comparing similarities between adults of some species and juveniles of others, under the assumption that all ophiuroids follow a common ontogenetic path with the same trajectory of shape changes. While this seems feasible, no detailed analysis has been performed yet. Ophiuroid postmetamorphic ontogeny has been studied in a small number of species (about 50 of the total >2,000), beginning with Ludwig [44,45], followed by Clark [46], Mortensen [40], Schoener [47,48], Webb & Tyler [49], Turner & Miller [50], and most recently two larger studies [41,51], most of these with a focus on external characters. Early ontogenetic stages are also known from the fossil record and they are similar to the typical postlarva of extant species [52]. The structure and development of internal skeletal elements in ophiuroid postlarvae, such as arm vertebrae, jaws and dental plates were examined by Matsumoto [32], but have not been studied with modern methods such as scanning electron microscopy.

The ophiuroid families Ophiuridae and Ophiolepididae (both families *sensu* [53]) are particularly rich in species with juvenile appearance, e.g. in the genera *Amphiophiura*, *Ophiomastus*, *Ophiomusium*, *Ophiozonella*, *Ophiopyrgus*, *Perlophiura* and others [43]. These are often quite small, with disc diameters of a few millimetres, and their skeleton consists of fewer elements than in the majority of extant species. Many live at great depths but it is unknown, whether the ecological conditions in the abyssal somehow further heterochronic development. The aim of this study is to assess whether paedomorphosis is the likely cause of the observed morphology of these species. We aim to establish a comparative basis for the detection of paedomorphic structures by defining juvenile states and comparing them to adult conditions and as far as possible to ancient states in fossils.

## Methods and Material

### Ophiuroid ontogeny

**Fig 1:**
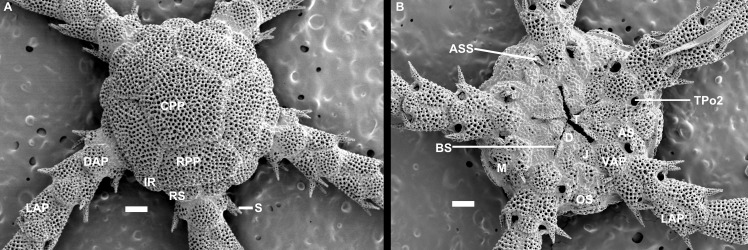
Skeletal morphology of Ophiuroidea postlarvae, *Ophiura sarsii*. **(A) dorsal aspect, (B) ventral aspect.** AS, adoral shield, ASS, adoral shield spine, BS, buccal scale, CPP, central primary plate, D, dental plate, DAP, dorsal arm plate, IR, interradial secondary disc scale, J, jaw = oral plate, LAP, lateral arm plate, M, madreporite, OS, oral shield, RPP, radial primary shield, S, arm spine, TPo2, second oral tentacle pore, T, tooth, VAP, ventral arm plate. Scale bars 0. 1 mm.

The here used terminology for the ophiuroid skeleton follows Sumida et al. [51], and Stöhr et al. [54]. In the species that have so far been examined, development follows a similar pattern, which we postulate here as the typical ophiuroid stages of juvenile development, based on our own examinations and other published data [21,41,47,48,51,55,56]. In most ophiuroid species, the dorsal disc of the smallest postlarva is formed by six plates, a centrodorsale and five radial primary plates (Fig 1), together known as the primary rosette, although exceptions where the rosette is lacking have been reported [41]. Radial shields and secondary disc scales appear at different stages during growth [41,51]. Dorsal arm plates are at first absent and develop gradually, starting on the proximalmost joints. Of the ventral arm plates the first one is always present from the youngest stages as part of the mouth frame, while the following develop gradually, but usually before the corresponding dorsal plate. Arm segments are relatively longer in juveniles than in adults, but adult distal arms are similar to juvenile arms, because the arms grow at the tip, proximal to the terminal plate, and thus the distal part is younger and less developed than the proximal part. Growth occurs first lengthwise, later widthwise. Tentacle scales are at first absent and appear at a rather late stage, after dorsal and ventral plates and independent of arm spine development.

In the oral frame of Ophiuridae and Ophiolepididae, at first the dental plate at the apex of the jaw bears a single tooth, and usually, a wide low lateral papilla termed buccal scale [55] is found at the oral plate. A second oral papilla is usually present in the adoral shield spine, at first usually spine-shaped and situated at the disc edge, next to the second oral tentacle pore, which is in early stages placed far from the mouth angle, into which it opens in adult Ophiuridae, but into which it is completely incorporated in Ophiolepididae. The oral shields are absent in the smallest postlarvae, except for the madreporite in some species. The madreporite in young stages can often be recognized by a cone-shaped protruding hydropore. Oral shields (including the madreporite) first appear at the dorsolateral edge of the disc [41,45,51] and move towards the mouth during ontogeny. The adoral shields that in adults frame the proximal and often lateral edges of the oral shields are present in early stages, since they are homologous to lateral arm plates. They obtain their specific shape and position during later ontogeny. In their juvenile state, the adoral shields reach around the oral shield, separating it from the arm, and the distal part of the adoral shields is placed at the lateral arm. During development the adoral shields shorten and in their most advanced state they are completely removed from the lateral arm, only bordering the proximal edges of the oral shield. The second tentacle pore is in young stages far from the mouth, but moves closer during ontogeny. It is associated with the adoral shields and the adoral shield spine, which turns into the distalmost oral papilla.

The order in which teeth appear seems to vary between taxa. Hendler [55] reported a series of teeth appearing at the same time in *Amphioplus abditus* (Verrill, 1871), Clark [46] described the first tooth to be the dorsalmost one, subsequent teeth forming ventralwards, whereas Stöhr [41] interpreted the first tooth as the ventralmost one, with subsequent teeth forming dorsalwards. Consequently, the dental plate may grow in either ventral or dorsal direction and tooth papillae in the Ophiotrichidae and Ophiocomidae may possibly form either in situ or by fragmentation of the ventral teeth. Clearly, this subject needs additional attention. Nevertheless, the dental plate in postlarvae is more or less round and cup-shaped, compared to the elongated, flat adult condition. Tooth sockets are simple openings or depressions in juveniles, but larger holes bordered by ridges or knobs in well developed adults.

### Taxon selection

We selected 40 species with more or less paedomorphic appearance from the families Ophiuridae (26 species) and Ophiolepididae (14 species) (Table 2). Most of the samples are housed in the collections of the Zoological Museum, Moscow State University (ZMMU), and the Swedish Museum of Natural History (SMNH). We also borrowed type specimens of paedomorphic species from Museum of Comparative Zoology, Harvard, Natural History Museum, London, US National Museum, Washington D.C., Zoological Museum, Amsterdam, Zoological Museum, Berlin, and Zoological Museum, University of Copenhagen, for additional data and as reference for correct identification of our material (Table 2). From type material we could gather mostly external characters, but from *Ophiomastus bulufonica* Tommasi, 1976, *Ophiomastus perplexus* Koehler, 1904 and *Ophiolepis impressa* Lütken, 1859 we received permission to dissect small arm pieces. To determine juvenile character states, we examined and dissected postlarvae of *Ophiura sarsii* Lütken, 1855 of the family Ophiuridae, *Ophiomusium lymani* Wyville-Thomson, 1873 of the family Ophiolepididae, and *Amphipholis squamata* (Delle Chiaje, 1828) of Amphiuridae. Also for several paedomorphic species, juveniles were available for examination. For comparison with a species with few or no paedomorphic character states, *Ophiocoma* (*Breviturma*) *krohi* Stöhr et al., 2013 was chosen, of which all skeletal structures have already been documented [57].

**Table 2:** List of studied species.

Family	Species name	Cat. no.	Stage	spms	DD [mm]	Parts
Ophiuridae s. Smith et al. (1995)	*Amphiophiura convexa*	SMNH 95535–38	J	5	3.2	ext, int
	*Amphiophiura latro*	SMNH 123492–93, 154489	A	3	8.8–9	ext
	*Anthophiura ingolfi*	SMNH 102176, 102182	A, P	3	1.9, 4.5, 4.84	ext, int
	*Aspidophiura cherbonnieri*	MNHN, no cat. no.	A	2	0.8, 1.2	ext
	*Bathylepta pacifica*	SMNH 99962–63, 100101, 100111	A, J	4	3–4, 2.24	ext, int
	*Ophiomastus bispinosus*	USNM E44367	A, J	2	5.4, 3.5	Ext
	*Ophiomastus bulufonica* T	USNM E11372	A?, P?	2	2.5, 0.95	ext, arm
	*Ophiomastus* cf. *bulufonica sp*. *1*	SMNH 102155, 102162, 102180	A?, P?	3	2.6, 1.9	ext, int
	*Ophiomastus* cf. *bulufonica sp*. *2*	SMNH 102163–64, 102166, 102173	A?, P?	3	2.5, 1.9	ext, int
	*Ophiomastus ludwigi*	USNM E7979	A?	1	3.1	Ext
	*Ophiomastus perforatus* T	ZMB 6778	?	1	1.1	Ext
	*Ophiomastus perplexus* T	ZMA U.EchO 2415 MCZ 3487	J,A	2,1	3.6, 3.24, 4.75	ext, arm
	*Ophiomastus primula* T	ZMB Ech 6777, SMNH 102177–79	A, J, P	2 + 4	2.0, 3.0 (T), 4.32, 2.0, 1.3	ext, int
	*Ophiomastus secundus* T	MCZ 407, SMNH 99903–04	A	1 + 3	3.0 (T); 3.2, 3.5	ext, int
	*Ophiomastus tegulitius* T	NHM 82.12.23.253, -254, -256, MCZ 418	A, J, P	3 + 1	3.8, 3.6, 2.1, 1.6	Ext
	*Ophiomastus tuberculata* T	USNM E11373	?	1	< 2	Ext
	*Ophiomastus tumidus*	ZMA 6560, MNHN EcOs 11449, SMNH 99529	A, J, P	8 + 19	1.7–4.6 (T); 3.5, 4.2, 1.85	Ext, int
	*Ophiomisidium irene*	SMNH 99952–56	A, P	10	2.68, 1.6	Ext, int
	*Ophiopyrgus planulatus* T	USNM 40928	A	1	3.5	Ext
	*Ophiopyrgus saccharatus* T	ZMB Ech 2487, SMNH 99530, 99533–34, 99542	A	1 + 3	6.5 (T); 5.0–5.1	Ext, int
	*Ophiopyrgus trispinosus* T	ZMA U.Ech O.2467, 2464, SMNH 127024–25	A	2 + 2	5.5, 5.8 (T); 3.1, 5.5	Ext
	*Ophiopyrgus wyvillethomsoni* T	MCZ 405, SMNH 100131, 102149	A	1 + 2	3.75 (T); 2.56, 3.1	Ext, int
	*Ophiotypa simplex*	SMNH 99539–41, 99543–46, 100117	A, J, P	9	4.2–4.4, 5.1, 2.25, 1.7	Ext, int
	*Ophiura sarsii*	SMNH 99925, 99936, 99938–29, 102151, 105835	A, J, P	12	22, 0.56, 0.86, 1.12	Ext, int
	*Ophiura serrata* T	ZMUC OPH-277	A	1	4.5	Ext
	*Perlophiura profundissima*	SMNH 99961, 99964, 99972, 100100, 102175	A, P	7	2.2, 3.0, 0.96	ext, int
Ophiolepididae	*Ophiolepis impressa* T	MZC-OPH-437	A	5	10–17	Ext, arm
	*Ophiozonella nivea*	ZMB Ech 5256	A	1	9.2	Ext
	*Ophiozonella clypeata* T	MCZ 318	A	1	10	Ext
	*Ophiozonella falklandica* T	ZMUC OPH-114, SMNH 99548–55, 99943	A, J, P	9 + 13	1.0, 1.1, 1.22, 1.4, 1.5, 1.65, 1.75, 2.25, 8, 10 (T); 5–6, 1.22	Ext, int
	*Ophiozonella longispina*	SMNH-111037-38	A	1	10	Ext, int
	*Ophiozonella novaecaledoniae* T	MNHN EcOs22118	A, P	2	2.12, 0.88	Ext
	*Ophiozonella projecta* T	ZMA U.Ech O. 2569, 2570, SMNH 108776, 111000	A	2 + 3	3.5, 3.7 (T); 4.3, 4.4	Ext, arm
	*Ophiozonella sincera* T	MNHN ECOS 22085	A, J	2	4.35, 3.12	Ext
	*Ophiozonella* sp. 2	SMNH 99901–02	A	2	3.7, 2.2	Ext
	*Ophiozonella* sp. 6	SMNH 99914, 99916–18, 99922–24, 99956	A, J	8	4.8, 4.2, 3.8, 2	Ext, int
	*Ophiozonella* sp. 7	SMNH 99915–16	A	3	2.1	Ext, int
	*Ophiozonella stellamaris*	SMNH 99547, 99945, 99950	A, P	6	2.9, 1.2, 1.4	Ext, int
	*Ophiozonella tessellate*	ZMB 5257	A	1	5	ext
*Ophiomusium* (new family required)	*Ophiomusium lymani*	SMNH 102150, 130490, 130493, 130498–500	A, J	2	24.3, 2.4	Ext, int
Amphiuridae	*Amphipholis squamata*	SMNH 99941–42, 99944, 102154	P	11	0.64	Ext, int
Ophiocomidae	*Ophiocoma krohi*	SMNH-Type-8534, 8535	A	1	13	Ext, int

Developmental stage, number of specimens per repository, size, and if external (no dissection) or internal parts examined (dissection of disc), or only dissection of arm. Species that included type material are marked with T. A, adult, DD, disc diameter, J, juvenile, P, postlarva (juveniles < 2 mm DD). The material can be located by its catalogue numbers at these repositories: MCZ, Museum of Comparative Zoology, Harvard, NHM, Natural History Museum, London, SMNH, Swedish Museum of Natural History, Stockholm, USNM, US National Museum, Washington D.C., ZMA, Zoological Museum, Amsterdam, ZMB, Zoological Museum, Berlin, ZMUC, Zoological Museum, University of Copenhagen.

### Preparations and measurements

Whole specimens were lightly bleached with household bleach NaOCl diluted 1:1 with tap water, to remove the outer integument. The dried specimens were then mounted on aluminium stubs with spray glue, examined in a Hitachi FE-SEM S4300 scanning electron microscope (SEM) and digitally photographed. Additional specimens were dissolved in concentrated bleach, the skeletal elements washed in tap water, and jaws, vertebrae, dental plates and lateral arm plates were mounted on aluminium stubs as above, examined and photographed in the SEM.

We analysed 29 characters from material specifically prepared during this study as well as from our image archives, and classified them as postlarva (= early postmetamorphic), juvenile (= intermediate between postlarva and adult) or adult (Table 3). Thus, the states as described in Table 3 are based on the respective ontogenetic stages in non-paedomorphic species, e.g. a primary rosette is not obvious (= same size as disc scales) in a fully developed dorsal disc, obvious but not dominating the disc (= larger than disc scales) in juveniles and dominating the disc (= no/few additional scales) in postlarvae. Juvenile state was assessed based on the conditions found in postlarvae with additional elements or shape changes, adult state was based on the terminal state of the ontogenetic trajectory of a well developed species from a different family than the studied group, because we suspect that all Ophiuridae and Ophiolepididae possess some paedomorphic characters. As an example, the medium sized postlarvae in Fig 1 have a primary rosette dominating the disc, weakly developed radial shields, separated dorsal arm plates along the entire arm, no ventral disc scales, a madreporite that differs from the other oral shields (by a clearly visible hydropore), the second oral tentacle pore far from the mouth slit. Weakly defined states are observed in early ontogeny when a structure has begun to form and its typical shape has not manifested itself yet. In these early stages, the edges of the skeletal element are fuzzy, any depressions, knobs or flanges are barely noticeable. Each state was assigned an integer value as a "paedomorphosis score", with zero denoting a non-paedomorphic adult state and higher numbers assigned to increasingly juvenile-looking characters. We follow the classification of the World Ophiuroidea Database [58].

**Table 3:** Character states used for scoring developmental/paedomorphic status.

Code	Character	adult = 0	juvenile = 1	postlarva = 2
A	Primary rosette	not obvious	not dominating disc	dominating disc
B	Dorsal disc scales	numerous	few	absent
C	Stereom (calcareous microstructure) structure	dense meshwork	open meshwork	
D	Radial shields	well developed	weakly developed	absent
E	Ventral disc scales	numerous	few	absent
F	Adoral shield	specific shape	similar to LAP	
G	Adoral shield spine	modified	spiniform	
H	2nd tentacle pore	inside oral slit	superficial	far from oral slit
I	Oral shield position	far from disc edge	close to disc edge	absent or dorsolateral
J	Oral shield shape	width > length	length > width	
K	Madreporite	as normal OS	differs from OS	with protruding cone
L	Oral papillae, lateral	several, differentiated	few, block-like	single
M	Oral plate	short	elongated	curved
N	Oral plate suture	not obvious	obvious	fragmenting
O	Dental plate	long, flat	short, convex	round, cup-shaped
P	Tooth sockets (or on external examination, number of teeth)	> 2	1–2	
Q	Genital plate adradial	well developed	weakly developed	absent
R	Genital plate abradial	well developed	weakly developed	absent
S	Proximal vertebrae	L ≤ H	L > H	
T	Vertebrae shape	entire	partially unfused	
U	DAP presence	along entire arm	only proximal	absent
V	DAP sequence	contiguous	separated	
W	DAP shape	as wide as arm	narrower than arm	
X	Arm spine articulation	well defined	weakly defined	absent
Y	Tentacle scale	along entire arm	only proximal	absent
Z	Tentacle scale shape	operculiform	spiniform	
AA	VAP	along entire arm	only proximal	absent
BB	VAP sequence	contiguous	separated	
CC	VAP shape	as wide as arm	narrower than arm	

Values are arbitrary, with 0 denoting fully developed adult states, higher numbers denoting less developed states as found in juveniles, and the least developed state as found in small postlarvae. When the difference between juveniles and postlarvae was small, we assigned just one juvenile value. A-L are external disc characters, M-T are internal characters and U-CC are external arm characters. DAP, dorsal arm plate, H, height, L, length, OS, oral shield, VAP, ventral arm plate.

**Fig 2:**
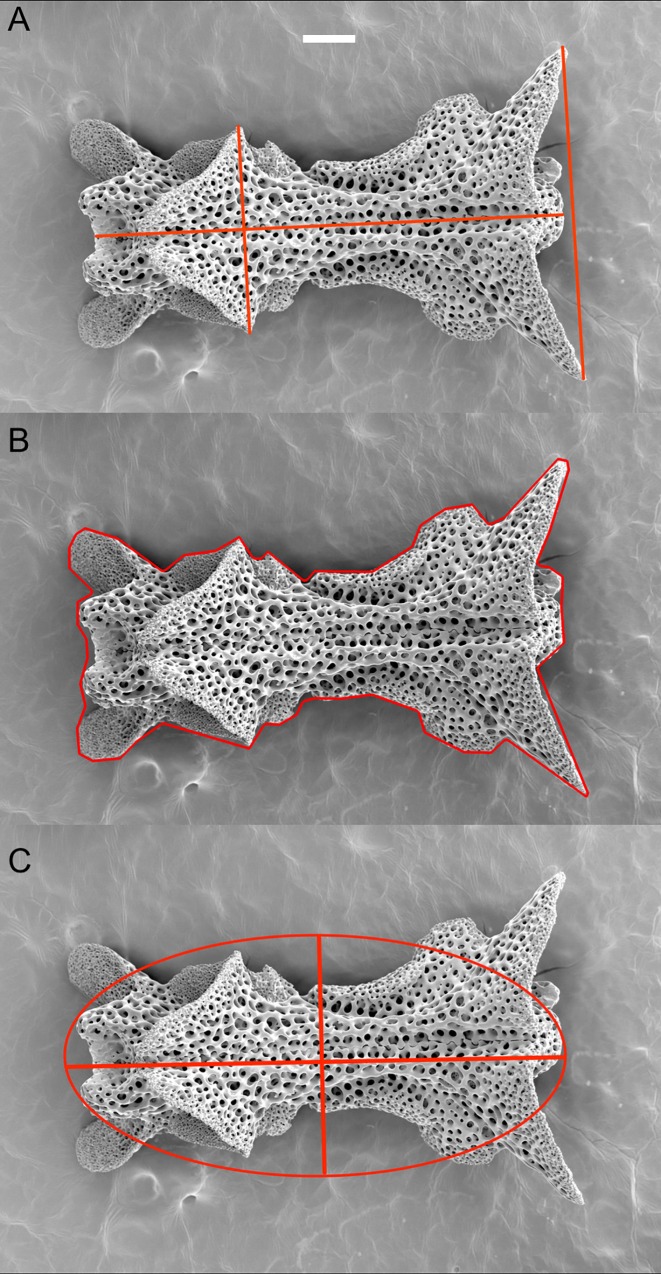
SEM images of a proximal arm vertebra of *Ophiozonella falklandica*, dorsal view. (A) Selecting a place to measure the length (horizontal line) and width (vertical lines) is difficult due to the complicated shape of the ossicle. (B) For a more standardized approach, in the software ImageJ a polygonal line is drawn around the shape of the ossicle. (C) Using a built in function of ImageJ an ellipse is then fitted to the ossicle; major (here length) and minor (here width) axes are measured automatically. Scale bar 0.1 mm.

The "paedomorphosis score" is admittedly subjective since continuously developing structures are difficult to delimit into well defined stages. Therefore we also developed a mathematical approach that can easily be reproduced. We selected arm vertebrae (from the proximal arm), oral plates, and dental plates, for morphometric measurements. These skeletal elements vary in their precise shape with flanges and knobs protruding to various degree, which makes it difficult to measure length and height or width at exactly the same spot (Fig 2A). Therefore, we decided to develop a standardized approach. We fitted an ellipse to these skeletal elements on the SEM images in the software programme ImageJ [59], measured the minor and major axes and calculated the quotient Major/Minor. This is a simple procedure in which the scale bar of the SEM image is used to calibrate the tool, then a polygon is drawn around the outline of the object (Fig 2B), a programme function is used to fit the ellipse and then the programme measures the axes of the ellipse (Fig 2C). In most cases, the major axis was the length of the structure, if not, we corrected for that. The resulting ellipses were rarely accurately parallel to the horizontal axis of the object, and we compared the values to measurements of straight lines placed on the object by eye. The absolute numbers between these methods differed, but the results in ordering the species according to the geometry of their skeletal elements were comparable. The ellipse method can more reliably be reproduced than the placement of a straight line arbitrarily across an irregularly shaped object. Thus we deemed the ellipse method more objective.

## Results

**Fig 3:**
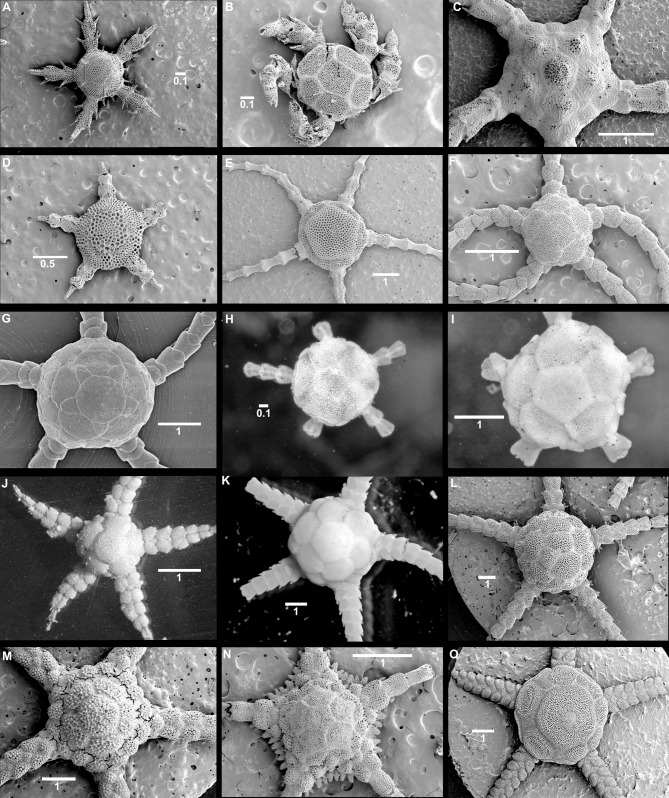
Examples of external morphology of postlarvae and paedomorphic adults. SEM images (except H-G). (A) Dorsal views of postlarvae of *Ophiura sarsii*, (B) *Amphipholis squamata*, (C) *Ophiomusium lymani*, and (D) *Perlophiura profundissima*, (E) adult *P*. *profundissima*, (F) juvenile *Ophiozonella stellamaris*, (G) adult *O*. *stellamaris*, (H) postlarva syntype of *Ophiomastus bulufonica*, (I) adult syntype of *O*. *bulufonica*, (J) postlarva syntype of *Ophiomastus tegulitius*, (K) adult syntype *O*. *tegulitius*, (L) adults of *Ophiomastus tumidus*, (M) *Ophiopyrgus saccharatus*, (N) *Ophiomisidium irene*, (O) *Ophiotypa simplex*. Scale bars in mm.

The morphology of the small postlarvae of *Ophiura sarsii*, *Amphipholis squamata* and *Ophiomusium lymani* concurs with previously published observations [51,41]. At the 2–3 arm joint stage (free joints beyond the disc), the disc is formed by six primary plates (one central and five radial, forming a rosette), no radial shields are evident, the arms are formed by joined pairs of lateral plates, lacking dorsal and ventral plates and they bear two spines (Fig 3A–3C). The dorsal and ventral arm plates form later, as do the radial shields. The postlarvae of *Perlophiura profundissima* Belyaev & Litvinova, 1972, *Ophiomastus bulufonica* Tommasi, 1976 and *Ophiomastus tegulitius* Lyman, 1878 also follow this general pattern. They can be recognized as juveniles by the thinner plates with more open meshed stereom, particularly obvious in the small postlarva of *P*. *profundissima*. The adults of these species possess small radial shields and small dorsal and ventral arm plates on proximal joints. In *P*. *profundissima* adults the central primary plate occupies a slightly larger part of the disc than in its postlarva. The radial primary plates separate its small radial shields. The stereom of all disc plates is denser than in the postlarva of this species, but still with larger pores than in other species. The arms are long and narrow, each segment is proportionately quite long, the dorsal arm plates are short, wide figure-eight shaped on top of the distalmost part of each arm joint. Ventral arm plates are equally small and placed at the distal end of each joint; on the distalmost joints both dorsal and ventral plates are lacking. The cup-shaped dental plate bears a single tooth.

Species with barely more than a primary rosette and small radial shields on the dorsal disc, few oral papillae and arm spines, with long arm joints and small dorsal and ventral arm plates, closely resemble postlarvae (Fig 3), apart from their larger size. The disc plates of *Ophiotypa simplex* are puzzling. Typical specimens seem to posses only large primary plates, but no radial shields. However, the large radial primary plates are articulated with the genital plates and may thus be fused radial shields, in which case the radial primaries may be lost. One of our specimens showed pairs of plates at two radii (Fig 3). These may either be fragmented radial primaries or unfused radial shields.

**Fig 4:**
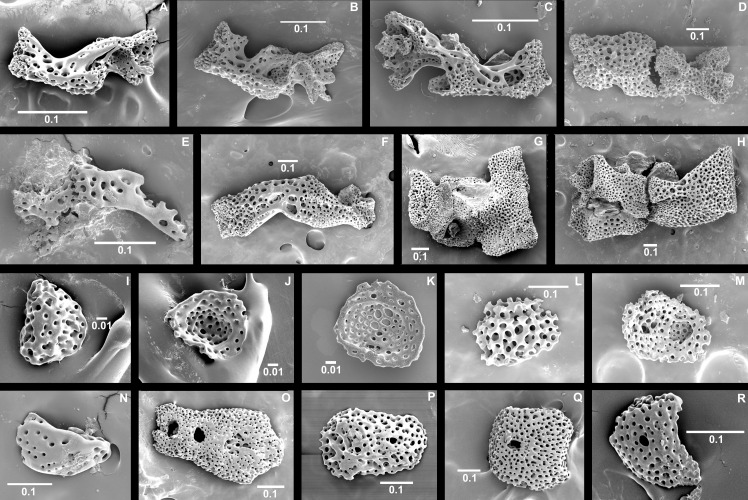
Examples of internal morphology of postlarvae and paedomorphic adults, oral plates and dental plates. SEM images. (A-H) Oral plates, (A) postlarvae of *Ophiura sarsii* at dd 560 μm, (B) same at dd 860 μm, (C) *Amphipholis squamata* at dd 640 μm, (D) *Ophiomusium lymani* at dd 2.4 mm, and (E) *Perlophiura profundissima* at dd 960 μm, (F) adults of *P*. *profundissima*, (G) *Ophiozonella stellamaris*, and (H) *Ophiotypa simplex*. Dental plate external (convex) or internal (concave) aspect of (I, J) *O*. *sarsii* at dd 560 μm, (K) *A*. *squamata* at dd 640 μm, (L, M) *O*. *lymani* at dd 2.4 mm, (N) adults of *P*. *profundissima*, (O) *O*. *stellamaris*, (P) *O*. *tumidus*, (Q) *O*. *simplex*, and (R) *Ophiozonella* sp. 7. dd, disc diameter. Scale bars in mm.

In postlarvae, jaws (oral plates), vertebrae (and corresponding lateral arm plates) are proportionately longer than in adults, confirming that growth occurs at first lengthwise, later widthwise (Fig 4). Each jaw half is composed of a proximal and a distal oral plate, which are firmly fused in the adults of most taxa. In juveniles however, a suture line can be observed and the plates may even separate when bleached, as we noticed in the juveniles of *Ophiomusium lymani* and the adults of *Ophiozonella falklandica*, *Ophiomastus tumidus*, *Ophiotypa simplex*, and an unidentified *Ophiozonella* sp. 7 (Fig 4). Juvenile jaws are also strongly curved, whereas adult jaws are straight. The dental plate at the tip of the jaws is an elongated flat plate in adults, but in postlarvae it is rounded and depressed cup-shaped, in the smallest stages with a socket for a single tooth (Fig 4). Some species with high total paedomorphosis scores, such as *O*. *falklandica*, are less paedomorphic for this character than others (e.g. *O*. *tumidus*, *O*. *simplex*, *Ophiozonella* sp. 7), possessing a flat, elongated dental plate with sockets for three teeth.

**Fig 5:**
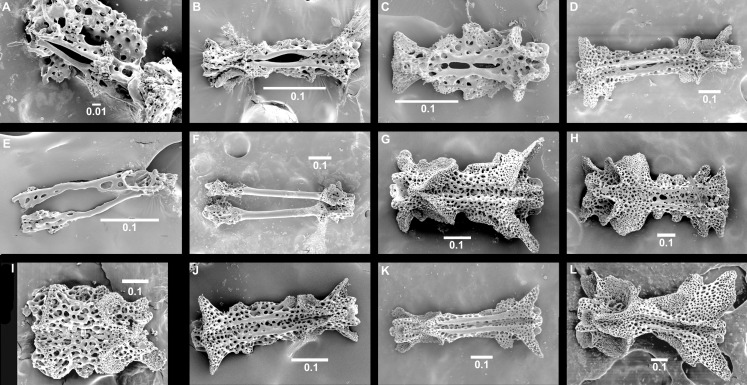
Examples of internal morphology of postlarvae and paedomorphic adults, arm vertebrae. SEM images. Postlarvae of (A) *Ophiura sarsii* at dd 560 μm, (B) same at dd 860 μm, (C) *Amphipholis squamata* at dd 640 μm, (D) *Ophiomusium lymani* at dd 2.4 mm, and (E) *Perlophiura profundissima* at dd 960 μm, (F) adults of *P*. *profundissima*, (G) *Ophiozonella stellamaris*, (H) *Ophiotypa simplex*, (I) *Ophiomastus tumidus*, (J) *Ophiozonella* sp. 7, (K) *Ophiomastus bulufonica*, and (L) *Ophiopyrgus saccharatus*. Scale bars in mm.

Each arm vertebra is formed by two ambulacrals as is evidenced in many taxa by a visible suture on the longitudinal midline of the vertebra. In juveniles, these halves are often incompletely fused, leaving an opening in the mid-section of the vertebra (Fig 5). In adult *Perlophiura* all vertebrae are almost completely unfused and easily separate at both ends (Fig 5). Interestingly, they are less fused than the vertebrae in small postlarvae of *O*. *sarsii* and *A*. *squamata*. The proximal and distal articulations of these vertebrae are similar to those in the postlarvae, less well defined than in adults and with open meshwork stereom, but clearly recognizable as zygospondylus. Three species of *Ophiomastus*, and *Perlophiura profundissima*, had longer and narrower vertebrae as adults than the postlarvae we compared with. The postlarva of *P*. *profundissima* has a less developed skeleton than postlarvae of comparable size of other species, which may indicate delayed development already at this early stage.

**Fig 6:**
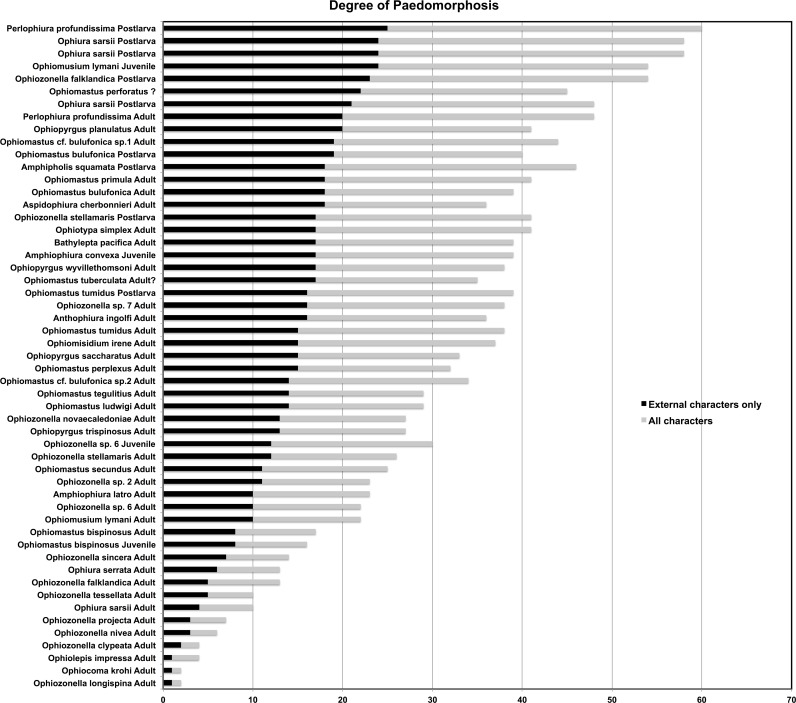
Total paedomorphosis scores for all species and ontogenetic stages. Sorted by external character score. 21 datasets do not include any internal characters (see Table 4).

**Fig 7:**
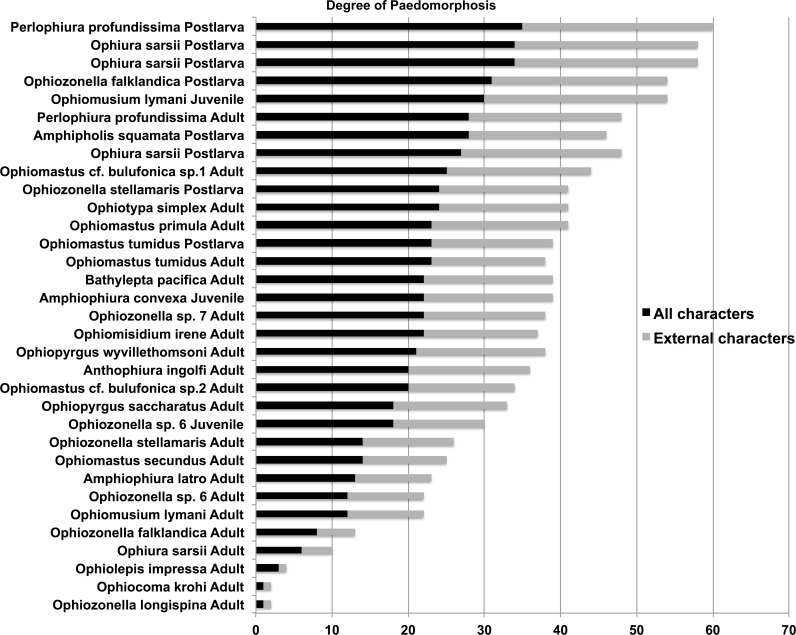
Total paedomorphosis scores for species and ontogenetic stages with both external and internal characters. Sorted by external character score. The general trend is the same as in Fig 6.

**Fig 8:**
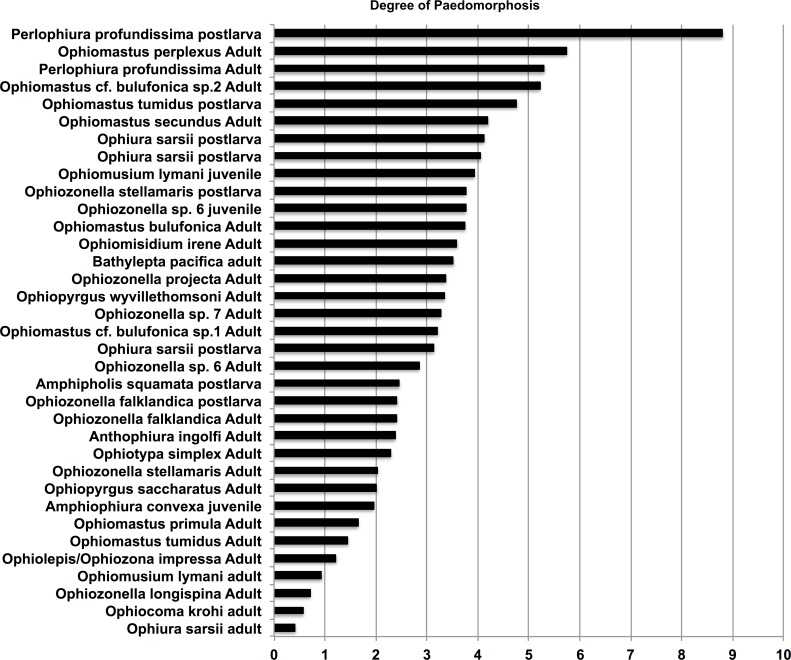
Degree of paedomorphosis by measurements of arm vertebrae. Only species for which vertebrae were available are included (see Table 4). X-axis values are length/width, sorted from low to high.

The smallest juveniles had the highest total paedomorphosis score and adult *O*. *krohi* had a total value of one (Table 4), which calibrates the score at both ends. Fig 6 shows all species and ontogenetic stages (54 datasets) arranged according to their external character score with bars for the total score including internal characters. Of these datasets, only 33 include internal character scores. Fig 7 shows only species with both internal and external character scores. Fig 8 plots the species for which vertebra measurements were available. Results are similar and demonstrate that paedomorphosis affects all parts of the ophiuroid skeleton. Of our 29 characters only eight are internal, but they contribute more or less proportionately to the total score. Not surprisingly, adult *Perlophiura profundissima* fall close to the postlarvae of other species and *Ophiozonella longispina* (H.L. Clark, 1908) is found at the weakly paedomorphic end of the curve. The highest score was assigned to the postlarva of *P*. *profundissima*, but this may be due to the fact that we were unsuccessful in isolating all target ossicles from the tiny postlarvae of other species (e.g. *O*. *sarsii*). According to our scores from external characters the most strongly paedomorphic species is *Perlophiura profundissima*. With regard to internal characters *P*. *profundissima* has the most paedomorphic vertebrae, and basic morphological patterns of both jaws and dental plate of *Perlophiura* are also very similar to corresponding postlarval festures of *Ophiura*, differing only insignificantly in some quantitative characteristics (Table 4).

**Table 4:** Paedomorphosis scores per species and ontogenetic stage according to Table 3.

Species name	Ont. Stage	A	B	C	D	E	F	G	H	I	J	K	L	M	N	O	P	Q	R	S	T	U	V	W	X	Y	Z	AA	BB	CC	∑	∑ ext
*Amphiophiura convexa*	Juv.	2	1	1	0	1	1	0	2	1	1	0	1	?	?	1	1	1	1	1	0	0	0	1	2	1	0	0	1	1	**22**	**17**
*Amphiophiura latro*	Adult	2	1	0	0	1	0	0	1	1	1	0	0	1	1	0	0	0	1	0	0	0	0	1	1	0	0	0	0	1	**13**	**10**
*Anthophiura ingolfi*	Adult	2	1	1	0	1	0	0	2	1	1	0	1	1	1	1	0	0	0	1	0	1	1	0	0	1	1	1	1	0	**20**	**16**
*Aspidophiura cherbonnieri*	Adult	2	1	0	0	1	0	0	2	1	1	0	2	?	?	?	?	?	0	?	?	1	1	1	?	1	1	1	1	1	**18**	**18**
*Bathylepta pacifica*	Adult	2	1	1	0	1	1	0	2	1	?	0	1	1	0	1	1	0	0	1	1	?	1	0	1	2	-	1	1	1	**22**	**17**
*Ophiomastus bispinosus*	Adult	2	1	0	0	1	0	0	2	1	0	0	0	?	?	?	1	?	?	?	?	0	0	0	?	0	0	0	1	0	**9**	**8**
*Ophiomastus bispinosus*	Juv.	2	1	0	0	1	0	0	2	1	0	0	1	?	?	?	?	?	?	?	?	0	0	0	?	0	0	0	1	0	**8**	**8**
*Ophiomastus bulufonica*	Adult	2	2	0	1	1	1	0	2	1	1	0	1	?	?	?	1	?	?	1	1	0	1	1	?	2	-	0	1	1	**21**	**18**
*Ophiomastus bulufonica*	Postl.	2	2	1	1	1	1		2	1	?	0	2	?	?	?	?	1	1	?	?	0	1	1	?	2	-	0	1	1	**21**	**19**
*Ophiomastus cf*. *bulufonica sp*.*1*	Adult	2	1	1	1	1	1	0	2	1	0	0	1	1	1	1	1	0	0	1	1	0	1	1	2	1	1	0	1	1	**25**	**19**
*Ophiomastus cf*. *bulufonica sp*.*2*	Adult	2	1	0	0	1	1	0	2	0	0	0	1	1	1	1	1	0	0	1	1	0	1	1	2	1	0	0	1	0	**20**	**14**
*Ophiomastus ludwigi*	Adult	2	1	0	2	1	1	0	2	1	1	0	0	0	?	?	1	?	?	?	?	0	0	1	?	0	0	0	1	1	**15**	**14**
*Ophiomastus perforatus*	?	2	1	1	2	2	1	1	2	1	1	0	1	?	?	?	1	?	?	?	?	1	1	0	?	2	-	1	1	1	**23**	**22**
*Ophiomastus perplexus*	Adult	2	1	0	0	1	1	0	1	1	0	0	1	?	?	?	?	?	?	1	1	0	1	1	2	1	0	0	1	1	**17**	**15**
*Ophiomastus primula*	Adult	2	1	0	1	1	1	1	2	1	1	0	0	1	1	1	1	0	0	1	0	0	1	1	2	1	0	0	1	1	**23**	**18**
*Ophiomastus secundus*	Adult	2	1	0	0	1	1	0	0	0	1	1	0	1	0	0	1	0	0	1	0	0	1	1	0	0	0	0	1	1	**14**	**11**
*Ophiomastus tegulitius*	Adult	2	1	0	1	1	1	0	1	1	1	0	1	?	?	?	1	?	?	?	?	0	1	1	?	0	0	0	1	1	**15**	**14**
*Ophiomastus tuberculata*	Adult?	2	1	1	1	1	?	?	?	1	1	0	1	?	?	?	1	?	?	?	?	1	1	1	?	2	-	1	1	1	**18**	**17**
*Ophiomastus tumidus*	Adult	2	1	0	1	1	1	0	2	1	1	0	1	1	1	1	1	1	1	1	1	0	1	1	?	0	0	0	1	1	**23**	**15**
*Ophiomastus tumidus*	Postl.	2	1	1	1	1	0	?	2	1	1	?	?	1	1	0	1	1	1	1	1	1	1	1	0	0	0	1	1	1	**23**	**16**
*Ophiomisidium irene*	Adult	2	1	0	0	1	1	0	2	1	1	0	1	1	1	1	1	1	1	1	0	0	1	0	?	1	0	1	1	1	**22**	**15**
*Ophiopyrgus planulatus*	Adult	2	2	0	2	2	1	0	2	1	1	0	1	?	?	?	1	?	?	?	?	0	1	1	1	0	1	0	1	1	**21**	**20**
*Ophiopyrgus saccharatus*	Adult	2	1	1	1	1	0	0	2	1	1	0	0	1	1	0	0	0	0	1	0	0	1	1	1	0	0	0	1	1	**18**	**15**
*Ophiopyrgus trispinosus*	Adult	2	1	1	1	1	0	0	2	1	1	0	1	?	?	?	1	?	?	?	?	0	0	1	?	0	0	0	0	1	**14**	**13**
*Ophiopyrgus wyvillethomsoni*	Adult	2	1	0	1	1	1	0	2	1	1	0	0	1	1	1	1	0	0	0	0	0	1	1	2	1	0	0	1	1	**21**	**17**
*Ophiotypa simplex*	Adult	2	1	0	2	1	1	0	2	0	0	0	2	1	1	1	1	1	1	1	0	0	1	1	2	0	0	0	1	1	**24**	**17**
*Ophiura sarsii*	Postl.	2	1	1	1	1	1	1	2	1	1	0	1	1	1	1	1	0	1	1	0	0	1	1	2	1	1	0	1	1	**27**	**21**
*Ophiura sarsii*	Postl.	2	1	1	2	1	1	1	2	1	1	1	2	1	1	1	1	2	2	1	1	2	-	-	2	2	-	2	-	-	**34**	**24**
*Ophiura sarsii*	Adult	1	0	0	0	0	0	0	1	0	1	0	0	1	1	0	0	0	0	0	0	0	0	0	0	0	0	0	1	0	**6**	**4**
*Ophiura sarsii*	Postl.	2	1	1	2	1	1	1	2	1	1	1	2	1	1	1	1	2	2	1	1	2	-	-	2	2	?	2	-	-	**34**	**24**
*Ophiura serrata*	Adult	2	0	0	0	0	0	0	1	0	0	0	0	?	?	?	1	?	?	?	?	0	0	0	1	0	0	0	1	1	**7**	**6**
*Perlophiura profundissima*	Adult	2	1	1	2	1	0	0	2	1	1	1	2	1	1	1	1	1	1	1	1	0	1	1	?	1	0	1	1	1	**28**	**20**
*Perlophiura profundissima*	Postl.	2	1	1	2	2	0	1	2	1	1	1	2	1	1	1	1	2	2	1	1	1	1	1	1	1	1	1	1	1	**35**	**25**
*Ophiolepis impressa*	Adult	0	0	0	0	0	0	0	0	0	1	0	0	0	?	?	1	?	?	1	0	0	0	0	0	0	0	0	0	0	**3**	**1**
*Ophiomusium lymani*	Juv.	2	1	1	1	1	1	1	2	1	1	1	2	1	1	1	1	0	1	1	0	1	1	1	2	1	0	1	1	1	**30**	**24**
*Ophiomusium lymani*	Adult	1	0	0	0	1	0	0	0	0	1	0	0	1	1	0	0	0	0	0	0	0	1	1	1	1	0	1	1	1	**12**	**10**
*Ophiozonella clypeata*	Adult	1	0	0	0	0	0	0	0	0	1	0	0	?	?	?	?	?	?	?	?	0	0	0	?	0	0	0	0	0	**2**	**2**
*Ophiozonella falklandica*	Adult	1	0	0	0	0	0	0	2	0	1	0	0	1	1	0	0	0	0	1	0	0	0	0	?	0	0	0	1	0	**8**	**5**
*Ophiozonella falklandica*	Postl.	2	2	1	2	2	1	0	2	0	1	0	3	0	1	1	1	2	2	1	0	1	1	1	2	1	0	0	1	1	**32**	**23**
*Ophiozonella longispina*	Adult	1	0	0	0	0	0	0	0	0	0	0	0	0	0	0	0	0	0	0	0	0	0	0	0	0	0	0	0	0	**1**	**1**
*Ophiozonella nivea*	Adult	1	0	0	0	0	0	0	0	0	1	0	0	?	?	?	?	?	?	?	?	0	0	0	?	0	0	0	0	1	**3**	**3**
*Ophiozonella novaecaledoniae*	Adult	2	1	0	1	1	1	0	2	1	1	0	1	?	?	?	1	?	0	?	?	0	1	0	?	0	0	0	1	0	**14**	**13**
*Ophiozonella projecta*	Adult	1	0	0	0	0	0	0	0	0	?	0	0	?	?	?	?	?	?	1	0	0	0	0	0	0	0	0	1	1	**4**	**3**
*Ophiozonella sincera*	Adult	2	1	0	0	0	0	0	0	0	1	0	0	?	?	?	?	?	?	?	?	0	0	1	?	0	0	0	1	1	**7**	**7**
*Ophiozonella sp*. *2*	Adult	2	1	0	0	1	1	0	0	1	1	1	0	?	?	?	1	?	?	?	?	0	1	1	0	0	0	0	1	0	**12**	**11**
*Ophiozonella sp*. *6*	Adult	2	1	0	0	1	0	0	2	1	1	0	0	1	0	0	0	0	0	1	0	0	1	0	?	0	0	0	1	0	**12**	**10**
*Ophiozonella sp*. *6*	Juv.	2	1	0	0	1	1	0	0	1	1	0	0	1	0	1	1	1	1	1	0	0	1	1	1	0	0	0	1	1	**18**	**12**
*Ophiozonella sp*. *7*	Adult	2	1	0	0	1	1	0	2	0	1	0	2	1	1	1	1	0	1	1	0	0	1	0	2	1	0	0	1	1	**819**	**16**
*Ophiozonella stellamaris*	Adult	2	1	0	0	1	0	0	2	1	1	0	1	1	0	0	0	0	0	1	0	0	1	0	0	0	0	0	1	1	**14**	**12**
*Ophiozonella stellamaris*	Postl.	2	2	0	1	2	1	0	2	1	1	0	1	1	0	1	1	1	1	1	1	0	1	0	1	0	0	0	1	1	**24**	**17**
*Ophiozonella tessellata*	Adult	2	0	0	0	0	0	0	0	0	1	0	0	?	?	?	?	?	?	?	?	0	0	1	?	0	0	0	0	1	**5**	**5**
*Amphipholis squamata*	Postl.	2	1	1	2	1	0	1	2	0	1	0	2	1	1	1	1	2	2	1	1	2	-	-	0	2	-	0	1	0	**28**	**18**
*Ophiocoma krohi*	Adult	0	0	0	0	0	0	0	0	0	1	0	0	0	0	0	0	0	0	0	0	0	0	0	0	0	0	0	0	0	**1**	**1**

Missing values coded as "?" could not be obtained due to lack of material. When a character that was also assessed for qualities was scored as absent, we coded the related characters as "-" to avoid scoring the same structure as absent multiple times. ext, external.

## Discussion

### Paedomorphosis and Phylogeny

Based on the above description of ophiuroid skeletal development, we can now analyse the status of adult morphologies. The primary rosette is present in most ophiuroids at an early stage, but in many species it is obscured during growth and often no longer distinguishable in adults. Hendler [55] commented for Amphiuridae that the primary plates are not regenerated when the disc is damaged (in contrast to radial shields and other structures), and concluded that the primary plates must be an ancient character that is retained in early ontogeny, but not necessary for disc formation in modern ophiuroids. The absence of typical primary plates in postlarvae of several extant species [41] supports this interpretation. A primary rosette was present in the earliest ophiuroids, as Hotchkiss [60] showed in a juvenile specimen of the extinct Palaeozoic order Oegophiurida (absent in adults), and retained through all of ophiuroid evolution, as evidenced by Jurassic postlarvae [52]. Thus, the existence of primary plates as such is a plesiomorphy that can be traced back to the very origin of the Ophiuroidea, but their presence in adult ophiuroids is a paedomorphic state. This is an important and still underestimated conclusion for the theory and practice of phylogenetic systematics (cladistics) since such an apparently plesiomorphic feature may reappear in heterochrony-driven descendant taxa many times, thus forming an apomorphic *pseudoplesiomorphy* [21]. Interestingly, primary plates seem to have been lost completely (not just in adults), several times independently on species level in several families [41]. This cannot be explained by heterochrony since secondary scales, and usually radial shields, are formed in these species, but possibly genes were switched off. If primary plates are not regenerated after disc damage, the question arises, how do strongly paedomorphic species that lack secondary scales repair their disc? Once their ontogeny has passed the stage of primary plate formation, do they keep the ability to switch the respective genes on again? Or do they switch on genes that are responsible for the formation of secondary scales, but which are not active during undisturbed growth? Since many of these species are found only at great depth (>2000 m) they are rarely collected and chances of finding regenerated specimens are slim; keeping them alive for laboratory experimentation is hardly possible. However, importantly, according to the available present and previous data [30,32,35] the paedomorphic morphology becomes evident in ophiuroids at depths below 1500–2000 m. This may be linked to low nutrient and energy potential of the bathyal and abyssal environments and corroborated by data from other groups, e.g. deep sea fishes [61]. The gene control behind ophiuroid development has barely been studied yet within a large comparative taxonomic framework, but may shed some light on these questions in the future since some pioneer studies on ophiuroid gene regulatory networks have appeared most recently [62].

Already Matsumoto [32] was aware of partially unfused vertebrae in some species and suggested a paedomorphic origin. The almost completely unfused vertebrae of adult *Perlophiura* may be evidence of extreme paedomorphosis in which the development is terminated earlier than the state found in typical Ophiuridae postlarvae. Fused vertebrae evolved from the left and right ambulacrals of Palaeozoic ophiuroid stem representatives [63] and therefore the unfused state could be plesiomorphic. Fused vertebrae occurred in the Ordovician to Devonian Oegophiurida [64]. The vertebrae of the Lower Carboniferous genus *Onychaster* were recently re-evaluated and classified as a special type of articulation, different from modern types [65]. Interestingly, *Onychaster* had quite short vertebrae, similar to adult *Ophiura sarsii*, suggesting accelerated development. Short vertebrae are also known from Oegophiurida [64], suggesting that fused, well developed vertebrae are a plesiomorphic state for Ophiuroidea. The long, lightly fused vertebrae of *Perlophiura* show the typical zygospondylus proximal and distal articulation faces of modern ophiuroids in the family Ophiuridae, which suggests that they are not descendants of a plesiomorphic lineage that never evolved fused vertebrae, but more likely development is indeed abbreviated by paedomorphosis. By the same argument, the partially fused vertebrae of other species examined here are also affected by paedomorphosis to various degrees.

Matsumoto [32] observed that in many species the lateral arm plates meet in the dorsal and/or ventral midline, the dorsal and ventral plates (if present at all) only covering part of the arm segment. He argued that the joined state of the lateral arm plates must be an advanced state, since the extinct Oegophiurida had an open ambulacral groove, which was covered over by ventral plates in later evolution. The lateral arm plate pairs were widely separated in these early forms. In juveniles the lateral arm plates are always joined, but may separate during later development, the opening being covered by ventral and dorsal plates. From this evidence we conclude that the joined state of lateral arm plates and widely separated or absent dorsal and ventral arm plates are not ancient, but paedomorphic states.

The position of the second tentacle pore inside the mouth is evidently the developed state, its position outside the mouth in adult specimens is paedomorphic. This suggests that the whole family Ophiuridae has a paedomorphic origin, whereas the genera (e.g. *Ophiolepis*, *Ophiozonella*, *Ophiomusium*) currently grouped in Ophiolepididae are not paedomorphic for this character. However, the genus *Ophiomusium* possesses several paedomorphic characters, such as presence of a primary rosette, few ventral disc scales, absence of most ventral arm plates, small dorsal arm plates, joined lateral arm plates, tentacle scales obvious only on few proximal joints, and elongated oral plates. These characters distinguish it clearly from the type genus *Ophiolepis* and a paedomorphic origin of the whole family Ophiolepididae is unlikely. More likely it is necessary to re-evaluate this family and possibly separate it into strongly paedomorphic (e.g. *Ophiomusium*, *Ophiosphalma*) and well developed species (e.g. *Ophiolepis*, *Ophiozonella*). The genus *Ophiomusium* was considered as a member of the Ophiuridae by Martynov & Litvinova [66], underlain by an at the time not yet completely published analysis of the spine articulation ridges and other micromorphological features (Martynov, 2010), but most recently its position as a close relative to the family Ophiuridae s.l. has been confirmed by a large transcriptomic analysis [67] and by a novel morphological phylogenetic analysis [68]. The present study thus provides additional evidence for a polyphyletic origin of Ophiuridae and Ophiolepididae as currently understood. There is however at least one species of *Ophiolepis* that may have evolved through progenesis, because its adults are considerably smaller than its congeners and their morphology corresponds to the juveniles of larger species [69]. The adults of several species of *Ophiozonella* examined in this study are moderately paedomorphic, but the generic type *O*. *longispina* has one of the lowest paedomorphosis scores.

The madreporite is lateral in early Ophiuroidea and in small postlarvae of modern forms, but ventral in all adults. The developmental trajectory seems to move from a clearly distinguishable plate with a cone shaped protrusion to a plate that more and more resembles a regular oral shield. Thus, a madreporite that can be distinguished from the other oral shields is a paedomorphic character.

Various, sometimes conflicting, classifications of heterochrony have been suggested and the usage and definition of terms such as neoteny and progenesis have changed over time, with more terms such as hypomorphosis being introduced [4,12,39]. Ideally, developmental rate changes should be compared between a species and its ancestor but ancestor-descendant pairs are rarely available for study and none are known for ophiuroids so far. The suggestion that paedomorphic ophiuroids are progenetic [34] was based on the small size of paedomorphic adults in comparison to non-paedomorphic adults. This approach is flawed, because there is no "general size" of non-paedomorphic ophiuroids. Both recent (e.g. *Amphipholis squamata*) and extinct faunas (Hotchkiss, personal communication) include diminutive species with well developed skeleton. Instead, we compare the size of individuals of similar developmental stage among closely related taxon groups. The evolutionary relationship between *Ophiura* and *Perlophiura* can be established by similarities of the arm spine articulations, lateral arm plates, and juvenile vertebrae. Terminal stages of *Perlophiura* reach a maximum disc diameter of 5 mm, whereas most species of *Ophiura* reach 10–15 mm disc diameter. The hypothetical non-paedomorphic ancestral taxa can be expected in the upper size range of *Ophiura*. *Perlophiura* possesses mostly postlarval characters. At a size of 2.2 mm dd it has longer and narrower arm joints than postlarvae of *O*. *sarsii* at 0.86 mm dd. In most species, postlarvae of more than 2 mm dd possess a greater number of skeletal elements and can be identified using keys for adult characters [41]. The so far largest early postlarva known is that of *Asteronyx loveni* [40,41], but it has a disproportionately large disc and short arms with short joints. *Perlophiura* thus appears to be an overgrown postlarva in which development is much slowed down, while growth continues. The same may be true for *Ophiotypa simplex*, and the here studied strongly paedomorphic species all seem quite large in relation to the status of their skeleton. The direct ancestor of these genera is not known and we cannot know if these species are larger than their ancestors, qualifying them for Gould's [4] definitions of neoteny or any of the later refinements of terms. Thus, our study provides additional evidence that rigid traditional distinction of several fixed heterochronic modes needs to be reconsidered, and using the more general term paedomorphosis is much more useful.

### Limitations

The approach used here successfully identified paedomorphic characters, but it has some limitations that may be approved upon with different techniques. The simple method of adding up score values was somewhat biased by the fact that not all target characters could be evaluated for all species. However, since it was not our intention to find the exact position of a species on a trajectory of heterochrony, the scores still suffice to show general trends.

The morphometric method was affected by the shape of the ossicles. The proximal vertebrae turned out to be the best indicator of juvenile/paedomorphic conditions. Oral plates (jaws) are probably undervalued by our method, because the fitted ellipse takes into account only the outline of an object, which works best with straight and relatively flat structures. Oral plates are strongly curved in young stages and a more correct measurement should at least measure the length along the curve, whereas we measured in a straight line from one end to the other. Measuring curves is difficult though, since SEM images are two-dimensional and curvatures or elevations are difficult to capture. The small size of the objects makes orientating them in a specific position extremely difficult, which may have lead to slight differences in positioning and measuring. The same applies to the dental plates that are cup-shaped in young stages and often almost round in outline. Even in vertebrae, there are projecting parts that affect the outline to variable degree depending on the exact orientation of the vertebra. For a statistically sound analysis, a larger number of specimens from each species should be examined. Methods of geometric morphometrics might be tried, but the aim of this study was not to find exact values and we do not believe that it is meaningful or possible to define the degree of paedomorphosis in that way. All skeletal elements have specific structures such as depressions, protrusions, flanges, excavations and other details. These may change shape during development, but are generally already present in young/paedomorphic specimens. They are also species specific and may obscure the developmental signals common to all species. Length to width ratios seem a simpler, but more robust measurement for the purpose of this study.

We cannot know the direct ancestors of the species studied here, and strictly speaking we cannot know if their development is really abbreviated in some mechanistic terms in comparison to the ancestral forms. Instead we propose that the observed morphologies are the result of complex developmental processes which may include a complicated mosaic of retardation and acceleration of morphological development underlain by changes in the gene regulatory networks. But ophiuroid development follows certain common paths as we and previous authors have demonstrated, and some species follow these paths much farther along than others to such a degree that the application of the term “paedomorphosis” for the majority of the studied cases is completely clear and unambiguous because not only some external features but also internal ones show such paedomorphic trajectories. These paedomorphic trajectories allow then the almost direct comparison of juvenile features of non-paedomorphic taxa with adult characters of paedomorphic ones, thus making the general pattern of phylogenesis less random and more predictable than has traditionally been concluded. Paedomorphic lineages may be quite old and indeed Ophiuridae and *Ophiomusium* are probably some of the oldest groups of Ophiuroidea. Thus one might argue that younger groups are peramorphic, displaying accelerated development. The fossil record however, seems to disprove this idea, since well developed forms are known from the oldest groups. Obviously, there is no limit for the appearance of paedomorphic taxa in a given group at any geological time and this is in concordance with the general conclusions on the evolution in the class Ophiuroidea [70].

## Conclusions

It is clear that species in which adults have vertebrae with a L:W ratio below 1 are well developed, whereas species with values close to 10 are strongly paedomorphic. Small juveniles (postlarvae) will be found at the upper end of this trajectory, but in some species even the smallest juveniles are more developed than adults of the most paedomorphic species.

The most strongly paedomorphic species are so similar to juveniles that it is difficult or impossible to decide on their ontogenetic stage without examination of the gonads. But, an indication of their paedomorphic nature can be found in their unusually large size in relation to the underdeveloped state of their skeleton. Some species of *Ophiomastus* may have been described erroneously, based on juvenile specimens. If their size falls within the typical range that correlates with their developmental state they are most likely juveniles and the validity of the species is in doubt. Such convincing evidence of almost direct influence of ontogenetic changes on adult patterns is of great importance for general taxonomic and phylogenetic practice. When strong heterochronic changes affect almost the whole skeletal system in ophiuroids, dramatic changes in adult morphology are produced, resulting in strongly paedomorphic appearance, as shown here. However, when heterochronic changes affect only some particular characters, like the adoral shield papillae, they produce less pronounced evolutionary effects, which may go unrecognized for a long time, resulting in the long-term neglect of a very common new species in an extensively studied region [21] thus directly affecting the core of taxonomic practice. This is especially relevant in the framework of the ontogenetic approach to taxonomy since our study revealed several unknown species in the genera *Ophiomastus*, *Ophiozonella* and *Perlophiura*. These will be described in a separate publication. The implications of our findings for the phylogenetic relationships between Ophiuridae and Ophiolepididae and within these groups will be analysed in a later study.

## Supporting Information

S1 File Paedomorphosis scores as verbatim. Same as Table 4 but in words instead of numbers.
(XLSX)

S2 File Morphometrics. Raw data, measurements of skeletal elements.
(XLSX)
